# A Gold-Standard for Entity Resolution within Sexually Transmitted Infection Networks

**DOI:** 10.1038/s41598-018-26794-7

**Published:** 2018-06-08

**Authors:** John Schneider, L. Philip Schumm, Maya Fraser, Vijay Yeldandi, Chuanhong Liao

**Affiliations:** 1 Department of Medicine, University of Chicago, Chicago, IL USA; 2 Department of Public Health Sciences, University of Chicago, Chicago, IL USA; 3 Chicago Center for HIV Elimination, University of Chicago, Chicago, IL USA; 4 Institute for Health Metrics and Evaluation, University of Washington, Seattle, WA USA; 5 Department of Medicine, University of Illinois at Chicago, Chicago, IL USA

## Abstract

Contact tracing for venereal disease control has been widespread since 1936 and relies on reported information about contacts’ attributes to determine whether two contacts may represent the same individual. We developed and implemented a gold-standard for determining overlap between contacts reported by different individuals using cell phone numbers as unique identifiers. This method was then used to evaluate the performance of using reported names and demographic characteristics to infer overlap. Cell-phone numbers, names and demographic data for a sample of high-risk men in India and their contacts were collected using a novel, hybrid instrument involving both cell-phone data extraction and Computer-Assisted Personal Interviewing (CAPI). Logistic regression was used to model the probability that a pair of contacts reported by different respondents were identical, based on the correspondence between their reported names and attributes. A discrete mixture model is proposed which provides predictions nearly as good as the logistic model but may be used in a new population without re-calibration. Despite achieving AUCs of 0.83–0.86, the low rate of true overlap among a very large number of contact pairs still results in a high rate of false positives. Next generation contact tracing calls for more archived or digital matching processes.

## Introduction

The use of contact tracing for venereal disease control has been widespread since 1936, when the Public Health Service first recommended that sex contacts of those infected with syphilis be found, notified, and interviewed for their own protection^1^. Since that time, contact tracing has become the standard of care and primary method of control efforts employed by local Public Health Departments for syphilis and Human Immunodeficiency Virus (HIV) across the United States^2,3^. Contact tracing has been utilized effectively to eradicate other infectious diseases such as smallpox and is a key strategic element in ongoing polio eradication efforts. Typically, the process of contact tracing in the context of HIV involves Disease Interventionist Specialists querying newly infected clients about their sex or drug contacts and then locating those contacts in the field to inform them that they have been exposed. Models indicate that this approach can be effective in reducing transmission^4,5^ and it may be cost-effective compared to other Public Health Department control efforts^6,7^. For these reasons this approach has been adopted in several other countries^8,9^.

Despite contact tracing’s potential for reducing disease transmission, the practical difficulties involved in identifying contacts limit its application. For example, reported names of sex or drug-using partners are often unreliable or ambiguous, due either to intentional concealment in an attempt to protect one’s privacy and/or that of others, or merely to a lack of knowledge when partners are not well-known or maintain multiple aliases. The latter is exacerbated by the increasing proliferation of social media and online communities where partners often meet without using or exchanging full names^10^. Even when partial name data are available, it can be difficult to determine whether contacts named by multiple newly-infected clients represent the same person (e.g., whether John Smith, age 29 named by Rick, is the same as J. Smith, age 26 named by Sam). If the criterion used for determining identical contacts is too permissive (e.g., if John Smith and J. Smith are assumed to be the same person based solely on the similarity of their reported names), then we risk missing individuals who may be infected and contributing to onward transmission. By contrast, using a strict criterion (e.g., refusing to treat John Smith and J. Smith as the same person because of the slight difference in their reported names, even if other corroborating information is available), increases the likelihood of “double-knocks” where the same individual is approached more than once. Such errors increase the risk of accidental disclosure, waste limited public health resources, and further alienate the community from the Public Health Service.

Several landmark contact tracing studies such as the Colorado Springs Study^11^ and many of its successors^12–14^ have attempted to use socio-demographic attributes (e.g., a partner’s gender or neighborhood) in conjunction with names to create entity resolution algorithms for locating identical individuals with a higher degree of certainty. Others have sought to improve accuracy by incorporating information on the structure of networks. For example, the likelihood that a pair of contacts named by two infected individuals are identical is higher if those individuals are themselves connected to one another^15^. Still others have employed time-intensive entity resolution processes which often require multiple interactions between researchers and study participants to validate findings^16^ While many of these studies have employed formal entity resolution algorithms, few have attempted to assess the performance of those algorithms, largely because there has been no gold-standard available with which to do so.

In this study we develop a gold-standard method for locating identical individuals among contacts using cell phone numbers as unique identifiers. We then use this gold-standard to estimate and evaluate two different models for entity resolution on the basis of names and other reported socio-demographic characteristics (Table 1).

**Table 1:** Example contact tracing data with phone number as the gold standard.

Entry	Name	Age	Race	Neighborhood	Marital Status	Phone #
1	Pat	35	White	West End	Married	555–1111
2	Patrick	35	White	Lakeview	Married	555–2222
3	Mark	25	Latino	Woodlawn	Married	555–3333
4	PJ	33	White	Lincoln Park	Single	555–2222
5	Fred	20	Black	Lawndale	Single	555–4444

Contact tracing information is not only subject to standard sources of reporting error, but also intentional error due to sensitivities surrounding sex partner information and disclosure. In this fictitious example, we see a potential equivalence between Entries 1 and 2, however according to the gold standard these are not the same individual because the phone numbers do not match. In contrast, Entries 2 and 4 are identical (i.e. the same person) as determined by the identical phone numbers—a match that may have been missed when following traditional matching algorithms.

The data were obtained from a sample of high-risk men who have sex with men (MSM) in India, a group that has had persistently high rates of HIV transmission. The ability to accurately identify individuals who may be in a particularly infectious period^17^ through contact tracing is a crucial building block toward eventual elimination of new HIV infections^18^. Improved contact tracing accuracy would not only facilitate efforts to reduce HIV transmission, but also other procedures aimed at improving health, combating terrorism, or enhancing social marketing.

## Methods

### Sample

Time Location Cluster Sampling (TLCS)^19,20^ was utilized with an existing sampling frame of Indian MSM^21^. Men were approached at different times of day through predefined intercepts in places where sex exchange occurs in a large South Indian City. Two-hundred and twenty-nine MSM respondents were recruited from 20 separate social venues for this study. The study was approved by the University of Chicago’s Institutional Review Board, and all recruitment and data collection procedures complied with relevant guidelines and regulations. Informed consent was obtained from all respondents.

**Figure 1:**
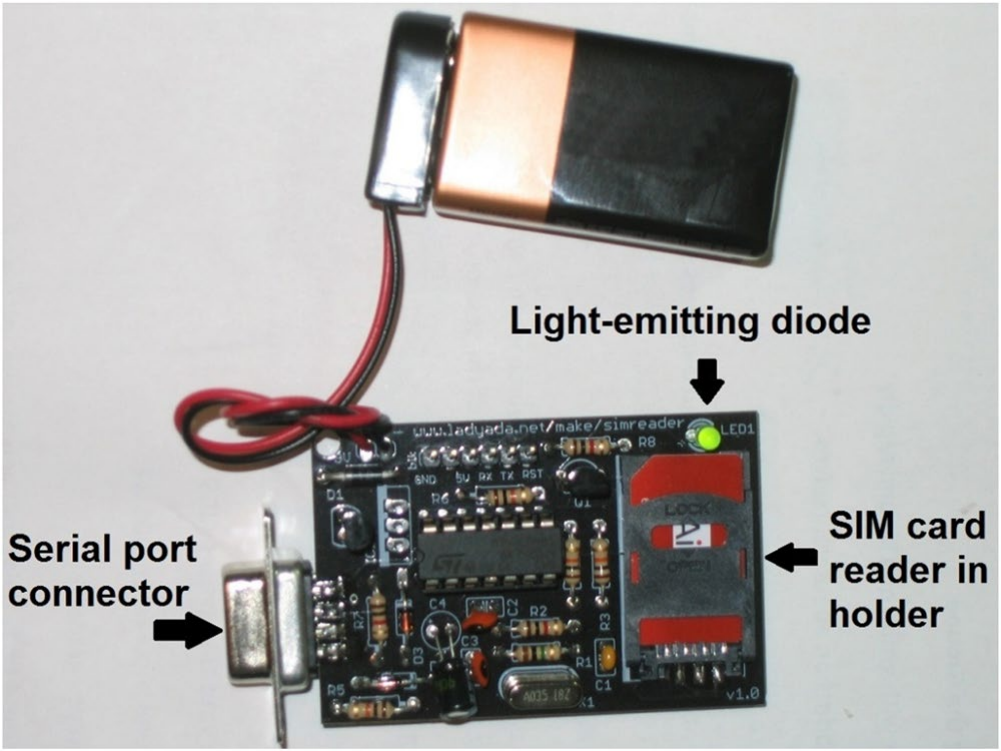
Subscriber Identity Module (SIM) card reader. The SIM card reader was assembled using a kit from Adafruit Industries (New York, NY). The card reader is operated by means of a program written using PySIM, a free open-source SIM card-reading software package.

Respondents’ contacts were exported electronically from their mobile phone address books using a custom SIM card reader built with the Arduino microcontroller^22^ and PySIM^23^, a free, open-source software package for SIM card management written in Python (Fig. 1). Contact names were then loaded automatically into a computer-based system designed to facilitate collection of additional information about individual contacts by an interviewer. Only those contacts identified by the respondent as being MSM were included in the analysis presented here. Among these, all possible *pairs* of contacts obtained from two different respondents were enumerated (i.e., pairs of contacts in which both contacts came from the same respondent were excluded), yielding 22,376,075 pairs. Eight-thousand and sixty-three pairs (0.04%) in which both contacts had the same phone number were considered to be *identical* (i.e., the same person), with the remainder considered to be *non-identical* (i.e., different people). To facilitate model estimation and interpretation, a random subsample of the non-identical pairs equal in size to twice the number of identical pairs was selected. The resulting 1:2 “case-control” sample of pairs was used for model fitting, however summaries are also presented for the entire sample.

### Measures

Each contact’s name and phone number were exported electronically from the respondent’s phone address book. The names varied from full names to only a first name, nickname or initials. First names (the majority of entries consisted of a first name only) were pre-processed by two native speaking experts who translated multiple versions of the same name to a standard form (e.g., Akeem, Akim and Akheem all became Akeem). The resulting set of first names were then matched using the Double-Metaphone phoneticizer^24^, allowing us to code each pair of contacts as either having the same first name, different first names, or being undetermined (if one or both contacts were identified by nickname or initials only). Respondents were also asked to describe each contact according to several demographic (age, neighborhood of residence (open-ended and classified into existing neighborhoods), religion, marital status) and sex behavior (MSM status and sex role (insertive, receptive or versatile)) characteristics, similar to what is collected by Disease Intervention Specialists in Public Health Departments. As with contact name, information on each characteristic was used to code each contact pair as either matching on that characteristic or not. Finally, a network measure of triadic closure was computed for each contact pair, indicating whether or not the two respondents who generated the pair of contacts were tied to each other (i.e., appeared in each other’s set of contacts) or not (i.e., neither appeared in the other’s set of contacts)^25^.

### Statistical Analysis

Stata 15 was used for all analyses. Logistic regression was used to model the probability of a pair of contacts from different respondents being identical (i.e., the same person, as determined by having the same phone number) as a function of whether both contacts in the pair had the same first name and were reported to be the same with respect to age, marital status, religion, neighborhood, and sex role^26^. Pairs for which first name matching was indeterminate were treated as non-matching with respect to name, while those with missing information on one or more of the other characteristics were excluded from the analysis. Because exact age may not be reported on accurately, cutoffs for determining a match from +/−1–10 years were tried, with +/−5 years being the most predictive (i.e., yielding the highest AUC); based on this, reported ages within 5 years of each other were considered matching. An initial model was fit using only the total number of matching characteristics (0–6, including first name) as a covariate. Because the characteristics vary in how socially salient they are (and therefore in the likelihood that they are reported on accurately), we then fit a model including each characteristic as a separate, binary covariate (matching versus not) together with the indicator of triadic closure. These two models were compared using both the Akaike and Bayesian information criteria, as well as the area under the Receiver Operating Characteristic (ROC) curve, or AUC. The second model was also fit to the subset of pairs where the primary relationship between respondent and contact was reported to be either “client” or “sexual partner” to determine whether it performed similarly among sexual contacts as compared to all MSM contacts.

While the logistic regression model is a standard approach for predicting a binary outcome, it has two disadvantages in this context. First, estimating the model requires knowledge of whether each pair is truly identical or not—knowledge provided here by our gold-standard, but in general not available. Since the parameters in the model are dependent on the nature of the population, the sampling procedure and the specific characteristics measured, it is unlikely that such a model estimated in one setting would be applicable in another. Second, the parameters have no direct substantive interpretation. Thus, while the model may be used merely to predict whether specific pairs are identical, it has limited value for describing the population and/or the process by which participants report on their contacts.

To overcome these limitations, we fit a second model to the data in which the true status of each pair (i.e., identical or not) is treated as a latent (unobserved) variable. Let p_1*j*_ be the probability that an identical pair matches on characteristic *j*. If all participants had perfect knowledge of their contacts’ characteristics and reported on them accurately, p_1*j*_ would equal one. Thus, the extent to which p_1*j*_ is less than one serves to measure both the completeness of participants’ knowledge of their contacts as well as their willingness to describe them honestly. In addition, let ${p}_{0j}$ be the probability that a non-identical pair matches on characteristic *j*. By contrast, this depends primarily on the distribution of that characteristic in the population, with characteristics that have fewer possible values (e.g., marital status) and a more uneven distribution across those values being more likely to match by chance alone than characteristics with a large number of possible values (e.g., age) and a more even distribution. Given these, we may write the marginal (i.e., overall) probability of a pair matching on characteristic *j* as

$${p}_{j}={p}_{1j}\theta +{p}_{0j}(1-\theta )$$

where *θ* is the overall probability that a pair is identical. This model is a discrete mixture model, also referred to as a latent class model^27^. The model is fit using maximum likelihood under the assumption of *local independence*, which means that conditional on whether the pair is identical or not, the probability of matching on one characteristic is independent of the probability of matching on another. Unlike the logistic regression model, the mixture model may be fit without knowledge of the pairs’ true status. In addition, its parameters have a direct interpretation and may therefore be used to assess the model’s validity based on substantive knowledge of the relative visibility of the characteristics.

Because estimating the AUC in the same sample used to fit a model tends to result in overestimates, we used k-fold cross-validation to obtain unbiased estimates of AUC for the logistic model^28^. Specifically, we performed 10-fold leave-one-out cross validation of the AUC averaging the 10 AUCs to get an overall estimate. This procedure was not necessary for the latent class model, since that model is fit without information on the true status of the pairs.

### Data Availability Statement

The datasets generated during and/or analysed during the current study are not publicly available due to highly sensitive network data and concerns over deductive identification of individuals, but individual level data are available from the corresponding author on reasonable request.

## Results

The distribution of characteristics among both the 229 MSM respondents and their contacts (n = 6,718) are shown in Table 2. The age distributions of respondents and their contacts were similar, with means of 27 (range 18–52) and 28 (range 14–68), respectively. MSM respondents were more likely to report themselves as being the insertive sex partner (27.4%) than their contacts (12.8%).

**Table 2:** Characteristics of MSM respondents and their MSM cell phone contacts.

	MSM respondents (n = 229)	Total MSM contacts (n = 6,718)
**Age, mean (SD)**	26.7 (6.8)	28.2 (6.8)
**Marital status, N (%)**		
Never married	147 (63.9%)	4332 (64.5%)
Married	58 (25.2%)	2197 (32.7%)
Separated/Divorced/Widowed	25 (10.9%)	179 (2.7%)
Don’t know	0 (0.0%)	10 (0.1%)
**MSM sex role, N (%)**		
Receptive	104 (45.2%)	3368 (50.1%)
Versatile	63 (27.4%)	2434 (36.2%)
Insertive	63 (27.4%)	861 (12.8%)
Don’t know/Missing	0 (0.0%)	55 (0.8%)
**Religion, N (%)**		
Hindu	190 (82.6%)	5480 (81.6%)
Muslim	25 (10.9%)	881 (13.1%)
Christian	15 (6.5%)	295 (4.4%)
Other/Don’t know	0 (0.0%)	62 (0.9%)

A logistic model predicting identical pairs using only the number of matching characteristics yielded an AUC of 0.80, while a model in which the coefficients for each characteristic were permitted to vary yielded a slightly higher AUC of 0.86. Matching first names had the largest effect with an estimated odds ratio considerably larger than those for the other characteristics (180.7 versus 1.6–3.5, Table 3). However, each characteristic when matching increased the odds of a pair being identical, adjusting for the other characteristics. In addition, being part of a triad (i.e., in which one respondent was also a contact of the other) also increased the odds of a pair being identical by an amount comparable to (or greater than) matching on each additional characteristic (except name). Results for the sex partner contacts only were similar, though matching sex role was less predictive of a pair being identical in this subgroup.

**Table 3:** Logistic regression models predicting gold-standard verified identical pairs.

Client characteristic	*All contact pairs* (*n* = *23,459*)^1^	95% CI	*Sex partner contacts only* (*n* = *2,787*)	95% CI
	Odds ratio		Odds ratio	
**First name**				
Match	180.7***	(135.6, 240.8)	131.7***	(68.7, 252.6)
Sex role	3.5***	(3.3, 3.8)	1.4	(1.0, 2.0)
Religion	3.1***	(2.8, 3.4)	3.6***	(2.2,6.1)
Neighborhood	3.0***	(2.7, 3.3)	2.5***	(1.6, 4.0)
Marital status	1.9***	(1.7, 2.0)	1.7**	(1.2, 2.5)
Age +/−5 yrs	1.6***	(1.5, 1.7)	1.7**	(1.2,2.6)
Part of a triad	3.0***	(2.7, 3.3)	4.0***	(2.6, 6.3)

^*^p < 0.05; ^**^p < 0.01; ^***^p < 0.001.

^1^All identical pairs together with a 2:1 random subsample of non-identical pairs.

**Figure 2:**
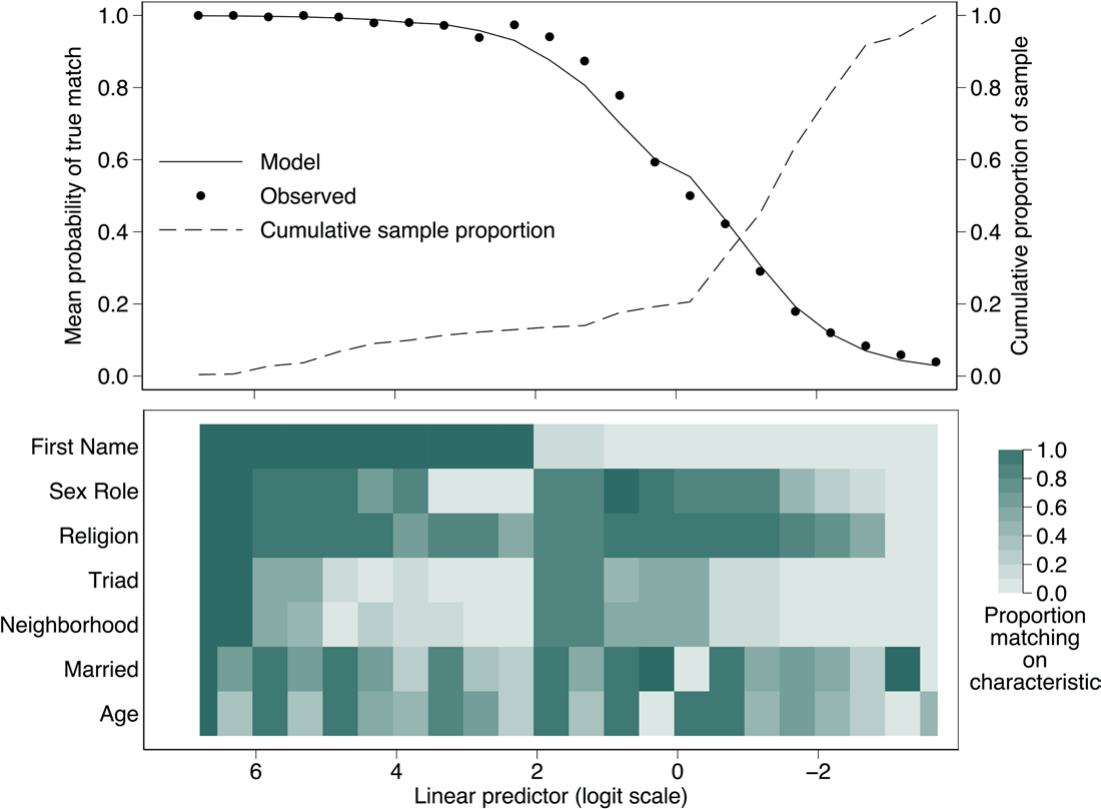
Heat map and plot demonstrating the probability of a true match given a set of client characteristics among randomly selected pairs of individuals from a large male sex network in South India.

Figure 2 shows the predicted and observed probabilities of a pair being identical for groups of pairs formed by splitting the linear predictor along its range (−3.7–7.3) into 22 intervals each 0.5 units wide. Each column in the bottom panel shows the proportion of the corresponding group that matched on each characteristic; the top panel shows the predicted and observed proportion of identical contacts for that group. Groups in which the proportion of identical pairs exceeded 0.9 consisted almost entirely of pairs matching on first name, though only a small number of these matched on neighborhood or were part of a triad (i.e., these are not required for a high likelihood of identity); if first name does not match, all other characteristics need to match to predict an identical pair with high probability. The lower predictive value of age and marital status is evident in the relative lack of pattern in the bottom two rows of the lower panel. The model fits relatively well (i.e., the observed proportions correspond well to the predicted proportions in the upper panel).

**Figure 3:**
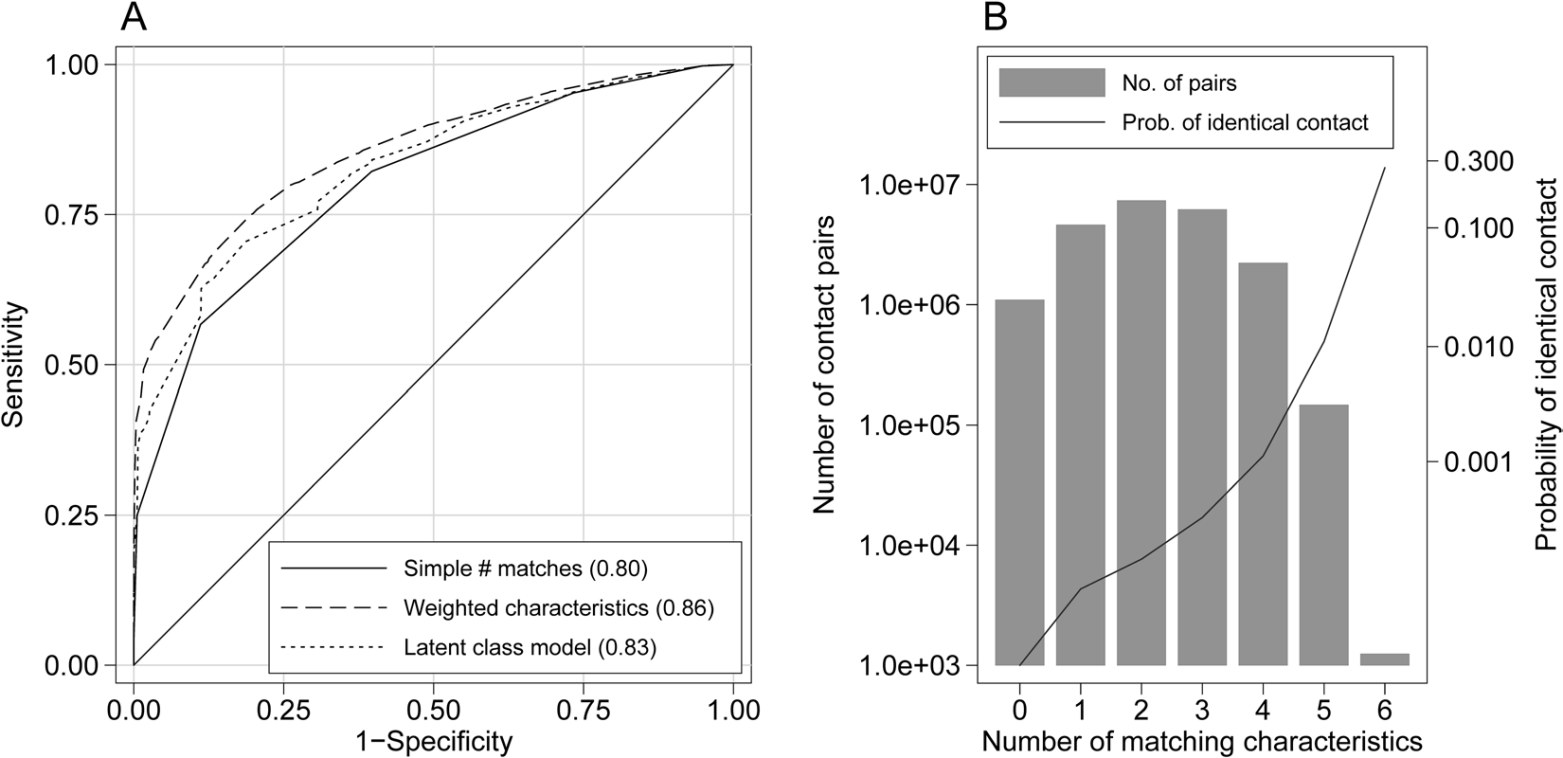
Sensitivities and specificities of contact matches. Panel A depicts the Area under the curve in parentheses (AUC) for likelihood of a contact match. Panel B highlights the number of pairs that match on given characteristics and resulting probability of having identical contacts.

Estimated parameters from the latent class model are shown in Table 4. The estimated proportion of identical pairs is 0.31, which is quite close to the true proportion of 1/3 in the case-control sample. Predictions from the model were overall nearly as accurate as those from the logistic model, with an AUC of 0.83 (Fig. 3A). However, the model under-predicted identical pairs among groups of pairs that matched on name but few if any other characteristics (Supplemental Fig. 1). Among identical pairs, first name and neighborhood were least likely to match—the former presumably due to the use of nicknames, initials, etc. (perhaps in some cases to intentionally conceal identity) and the latter due to its relative lack of social visibility and the ambiguous and overlapping ways in which neighborhoods are often defined. Among non-identical pairs, religion is estimated to match 68% of the time due to the overwhelmingly Hindu population (i.e., two contacts selected are random are both likely to be Hindu and therefore to match on religion). Age, marital status and sex role are likely to match by chance approximately 50% of the time, while first name and neighborhood are very unlikely to match by chance alone.

**Table 4:** Estimates from latent class model of characteristic matching fit to sample of identical and non-identical pairs (n = 23,459)^1^.

	*Class*	
	Identical pair	Non-identical pair
Estimated class proportion	0.31	0.69
***Class-specific probability of covariate match***		
First name	0.35	0.03
Sex role	0.73	0.40
Religion	0.93	0.68
Neighborhood	0.27	0.05
Marital status	0.83	0.49
Age +/−5yrs	0.78	0.48

^1^All identical pairs together with a 2:1 random subsample of non-identical pairs.

Figure 3B shows the effect of scaling up to the full sample of pairs on the accuracy of predictions based on matching characteristics. Even among those pairs that matched on all 6 characteristics (representing a small fraction of the total sample), the proportion of identical pairs is only 0.3; for those with 5 matching characteristics the proportion of identical pairs drops to 0.01 (though this is higher if name is one of the 5).

## Discussion

There are several important aspects of this work relevant for HIV elimination efforts. First, we describe a new approach to enumerating contacts and resolving their identity across respondents using names and phone numbers exported from mobile phones. By loading these automatically into a CAPI system, we are then able to obtain additional information about each contact via standard interview techniques. This hybrid approach is just one way that digital technology can be used to augment contact tracing efforts. Next generation contact tracing will require continued development of new methods for collecting digital information as well as for utilizing such information effectively^18^. For example, digital identities based on usernames, email addresses or phone numbers are becoming more relevant, and can be used to establish an identity match with greater confidence than with names alone. However, since people often maintain multiple online identities (or aliases), methods for collapsing over these different identities will be required. In addition, fully leveraging such information requires linking it to archived data such as those from Facebook or dating/hook-up apps, and this raises both technical and privacy issues that need to be addressed. Despite such obstacles, digital information promises to allow more accurate and rapid identification of core groups or key superspreaders within ongoing outbreaks. This is particularly important as the HIV epidemic begins to move toward active outbreak investigations rather than passive surveillance efforts.

By obtaining a gold standard for contact identity resolution, we are able for the first time to assess the accuracy of predictions based on reported characteristics traditionally collected by local health departments such as age, name and neighborhood. We find that a model in which the weights (i.e., regression coefficients) are permitted to vary across characteristics does indeed provide somewhat better predictions than a simple count of matching characteristics, owing to differences in the population distribution of the characteristics (and therefore in the likelihood that they will match for a randomly-selected pair) as well as in their social visibility. In addition, we find that including the network structure in which a pair of contacts is embedded can increase predictive power; specifically, pairs that form a closed triad (if identical) are more likely to be identical, and this is as predictive (and in some cases even more so) as matching on each additional characteristic (except for name). Future work might consider incorporating additional structural information, as well as information that may be related to network structure, such as where/when respondents are recruited—an approach increasingly possible with geospatial application data^29^.

A fundamental limitation with any model that requires calibration (e.g., a logistic regression model, machine learning models) is that it requires having a large enough dataset with a gold standard, as we obtained here, to build and test the model. Models developed in one population may require recalibration for use in others. For example, we observed that matching sex role was less predictive among sex partner contacts, a difference consistent with previous work^30^. The latent class model we propose here does not require previous calibration and performed nearly as well as the logistic model. Moreover, its parameters are directly interpretable in terms of the accuracy with which respondents are able and/or willing to report on their contacts characteristics.

This work highlights the main problem with using matching characteristics to predict contact identity in a large network; specifically, in a large network the true proportion of identical contacts will be low, and this combined with a high rate of matching by chance results in a large rate of false positives (i.e., predicting that two contacts are identical when they are not). This is especially problematic in the case of contact tracing, since failing to reach people at risk is a more serious error than “double-knocks” (i.e., contacting the same person twice). Overcoming this would require obtaining a larger number of characteristics and/or characteristics with a higher degree of uniqueness (to reduce the rate of matching by chance). However, these may turn out to be more difficult to obtain than simply obtaining identifiers directly (e.g., phone numbers or online identifiers).

We acknowledge several limitations to the analyses here that might be addressed in future work. First, we excluded pairs with missing data on one or more characteristics (except for name), in part because the proportion of missing data in this case was quite low. However, missing data may be more common in other situations, and one may wish to generate predictions for pairs with partial information. It should also be noted that names exported from electronic contact lists (as done here) may be more or less accurate and complete than names reported directly by respondents. Second, although we classified each pair as either matching or not matching for each characteristic, it is actually possible to quantify the degree of matching for items such as name and age, and it is possible that by utilizing this more detailed information we may improve our predictions. In addition, machine learning methods may be useful in this context, and should be explored. Finally, we recognize that our approach may not be appropriate for all contexts and populations.

In sum, advanced network tracing enhances the entire contact tracing enterprise. Inability to reach specific infectious network members limits our ability to identify clusters of cases where intervention is needed. Additionally, the alienation of individuals by public health departments through “double-knocks” can further limit efforts to link potentially at-risk community members to health screening and other treatment services. We must strengthen the public health service as the epidemic stabilizes in many contexts and rebound epidemics^31^ become the next front in HIV elimination efforts.

## Electronic supplementary material

Supplemental Figure 1
