# Supplementary material for: A Gold-Standard for Entity Resolution within Sexually Transmitted Infection Networks
Source: Sci Rep. 2018 Jun 8;8:8776. doi: 10.1038/s41598-018-26794-7 (PMC5993735; doi:10.1038/s41598-018-26794-7)
Supplement: Supplementary file 1 — Supplemental Figure 1 [file 41598_2018_26794_MOESM1_ESM.pdf]

A Gold-Standard for Entity Resolution within Sexually Transmitted Infection Networks

*John Schneider[1,2,3]
L. Philip Schumm[2]
Maya Fraser[4]
Vijay Yeldandi[5]
Chuanhong Liao[2]

1. Department of Medicine, University of Chicago, Chicago IL
2. Department of Public Health Sciences, University of Chicago, Chicago IL
3. Chicago Center for HIV Elimination, University of Chicago, Chicago IL
4. Institute for Health Metrics and Evaluation, University of Washington, Seattle WA
5. Department of Medicine, University of Illinois at Chicago, Chicago IL

Original Research Article

Corresponding Author:
John Schneider
5841 South Maryland Avenue, MC 5065
Chicago, IL 60637
773-702-8349
Jschnei1@medicine.bsd.uchicago.edu

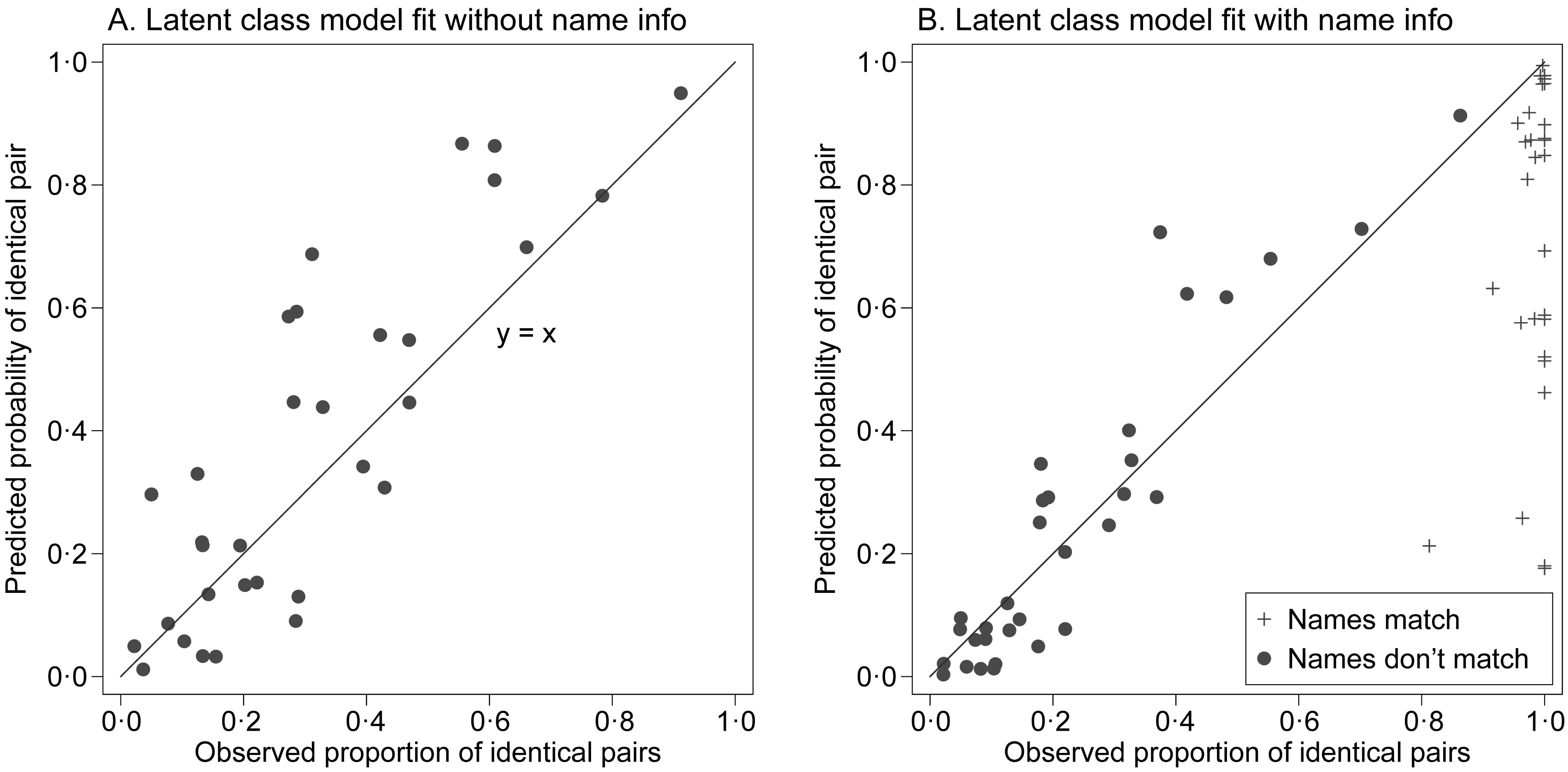
A. Latent class model fit without name info
B. Latent class model fit with name info
Predicted probability of identical pair
Observed proportion of identical pairs
y = x
1·0
0·8
0·6
0·4
0·2
0·0
Names match
Names don't match
